# Physiological and Transcriptome Analyses Offer Insights into Revealing the Mechanisms of Red Tilapia (*Oreochromis* spp.) in Response to Carbonate Alkalinity Stress

**DOI:** 10.3390/antiox14091112

**Published:** 2025-09-13

**Authors:** Wei Ye, Wen Wang, Jixiang Hua, Dongpo Xu, Jun Qiang

**Affiliations:** 1 Wuxi Fisheries College, Nanjing Agricultural University, Wuxi 214081, China; linda1995king@163.com (W.Y.); wangwen@ffrc.cn (W.W.);; 2 Key Laboratory of Freshwater Fisheries and Germplasm Resources Utilization, Ministry of Agriculture and Rural Affairs, Freshwater Fisheries Research Center, Chinese Academy of Fishery Sciences, Wuxi 214081, China

**Keywords:** red tilapia, NaHCO_3_-induced alkaline stress, tissue damage, physiological chemistry, RNA-seq

## Abstract

The utilization of saline–alkali water resources presents a promising approach for freshwater aquaculture. Red tilapia (*Oreochromis* spp.) exhibits moderate salinity tolerance, but its adaptation mechanism to alkaline conditions remains poorly understood. In the current study, five alkaline carbonate concentrations in a 60-day chronic stress experiment on red tilapia were evaluated. The experimental design included a control group (CA0, 0 mmol/L) and three treatment groups (CA10, 20 mmol/L; CA30, 30 mmol/L; and CA40 40 mmol/L). The results indicated that at alkaline carbonate concentrations exceeding 20 mmol/L, the gill filaments exhibited curling and deformation, the hepatocytes displayed migration, and tissue damage increased significantly. The gill’s antioxidant capacity initially decreased and then increased, with severe gill injury in the CA40 group, leading to significantly reduced levels of SOD, CAT, and GSH-PX compared to the CA40 group (*p* < 0.05). Conversely, the enzymatic activities related to energy metabolism showed an opposite trend under alkaline carbonate stress. The transcriptome analyses of gill tissues across five groups identified significant alterations in key pathways, including the metabolic process (endocytosis, focal adhesion, PI3K−Akt signaling pathway, MAPK signaling pathway, and Citrate cycle (TCA cycle)), and immune responses (mTOR signaling and NOD−like receptor signaling pathways). Additionally, we screened 13 differentially expressed genes (DEGs) as potential regulators of alkaline stress and validated their expression levels using quantitative real-time PCR (qPCR). This study preliminarily elucidated the molecular mechanism of red tilapia in the physiological regulation process under chronic alkaline stress, and offers a theoretical foundation for breeding programs aimed at developing alkali-tolerant strains for aquaculture in alkaline water environments.

## 1. Introduction

Land salinization has become an increasingly prominent global challenge, primarily due to poor freshwater management, drought, and inefficient irrigation practices [1]. China has 99,130 km^2^ of saline–alkali land, of which 45,870 km^2^ are low-lying saline–alkali water areas, accounting for 55% of the total lake area in the country [2,3,4]. In China, low-salinity saline–alkali water areas, with a salinity of approximately 10 mmol/L, are being developed and utilized by transplanting and domesticating conventional freshwater and seawater aquatic economic species. However, most medium- and high-salinity saline–alkali water areas with salinity levels exceeding 10 mmol/L remain barren and idle. Nevertheless, many medium- and high-salinity saline–alkali lakes worldwide still support certain fish species that have long inhabited and formed two dominant populations. For example, the naked carp (*Gymnocypris przewalskii*) [5] in Qinghai Lake (TA: 29 mmol/L), and Waleck’s yarok fish (*Leuciscus waleckii*) and crucian carp (*Carassius auratus*) [6] in Dali Lake, Inner Mongolia. Utilizing saline–alkali water for aquaculture instead of freshwater could significantly reduce freshwater consumption while supporting sustainable aquaculture development in affected regions. However, the high alkalinity, elevated pH, and complex ionic composition of saline–alkali water pose serious challenges to aquatic organisms [7,8,9]. Studies indicated that fish in such environments often experience osmotic imbalance, leading to disruptions in respiratory metabolism and immune suppression, as well as impaired growth, development, and reproduction [6,10,11]. Therefore, understanding the adaptation mechanisms of fish to alkaline conditions and identifying the saline–alkali-tolerant species are prerequisites for advancing saline–alkali aquaculture.

When aquatic animals are exposed to alkaline water, they experience physiological alterations in their metabolism of ammonia and nitrogen metabolism, osmotic regulation, and acid-base balance [2]. The gills, as key respiratory and excretory organs, play a crucial role in ion transport, ammonia excretion, and acid-base homeostasis [12,13]. Wood et al. reported that high alkalinity primarily disrupts osmoregulation and ion exchange in fish gills and cell membranes, impairing the acid-base buffering system and increasing the mortality risk [1,14]. Under alkaline conditions, fish experience elevated metabolic rates, raising internal ammonia levels. Concurrently, high external alkalinity inhibits ammonia excretion, leading to ammonia accumulation and potential toxicity—a major cause of death in highly alkaline waters. For example, Lahontan Cutthroat Trout (*Oncorhynchus clarkii henshawi*) in Pyramid Lake (pH 9.4) face such challenges [1,15]. Only a few species, such as *Leuciscus waleckii*, *Oreochromis niloticus*, and *Carassius auratus gibelio* [16,17,18], thrive in a highly saline–alkali water environment. To cope with carbonate alkalinity, fish employ adaptive mechanisms, including gill acid-base regulation, CO_2_ excretion, and ion/osmotic adjustments, enabling them to mitigate alkalinity fluctuations and survive in harsh conditions [12].

Adaptation to and tolerance of alkaline conditions have been extensively studied in teleost fish. These species regulate osmotic balance through integrated ion and water transport mechanisms within their osmoregulatory systems [19]. Euryhaline fish exhibit remarkable adaptability in saline environments, maintaining a stable ionic composition and osmolality via morphological, cellular, and physiological modification of their gills [19,20]. Similarly, *Cyclina sinensis* adapts to salinity stress involving gill osmoregulation, energy metabolism, and immune responses [21]. Several studies have examined the effects of salinity on fish growth in species such as *Mugil liza* [22], *Trachinotus ovatus* [23], and *Oreochromis niloticus* [24]. Some species demonstrate enhanced growth in both low and high salinity environments.

The Guidelines for the Development and Utilization of Saline-Ash Aquaculture, issued by the Ministry of Agriculture and Rural Affairs in 2021, clearly state the goal to” reduce salt through fish farming, treat alkalinity with fish farming, to improve the ecological circulation of water bodies by breeding fish tolerant to saline-ash conditions, gradually lowering the pH and salinity of saline-ash water while generating economic value”. To date, many studies involving fish species such as *Carassius auratus* (TA = 60 mmol/L) [6], *Micropterus salmoides* (45 mmol/L) [2], *Ctenopharyngodon idella* (45 mmol/L) [4], and *Siniperca chuatsi* (20 mmol/L) [25] have employed alkalinity levels that are higher than those typically found in natural water bodies. Furthermore, Liu et al. (2023) found that under long-term alkalinity stress in *Oreochromis niloticus*, the acceptable concentration of carbonate alkalinity for the fish reached 35.7 mmol/L [26]. Red tilapia (*Oreochromis* spp.), a euryhaline species, exhibits greater tolerance to saline–alkaline conditions than most freshwater fish. This adaptability makes it an excellent candidate for saline–alkaline aquaculture [27]. Due to its rapid growth, robust adaptability, high nutritional value, and strong disease resistance, red tilapia has become a key species in large-scale aquaculture in China [28]. Therefore, this study aims to assess the effects of different carbonate alkalinity levels (CA0, CA10, CA20, CA30, and CA40 mmol/L) on red tilapia, with a particular focus on their metabolic capacity, antioxidant capacity, and gill morphology. Additionally, transcriptome sequencing of gill tissues was also employed to elucidate the molecular regulatory mechanisms underlying alkalinity stresses responses. By integrating physiological and transcriptomic analyses, this study aimed to enhance our understanding of carbonate alkalinity on red tilapia. Additionally, it seeks to provide a foundation insights into the molecular strategies fish employ to cope with saline–alkaline stress.

## 2. Materials and Methods

### 2.1. Sample Culture and Experimental Design

The experimental larvae were raised for 60 days in the fish farming workshop (Building F) of the Freshwater Fisheries Research Center of the Chinese Academy of Fishery Sciences (Wuxi, China). A total of 375 individuals of *Oreochromis* spp., with an initial average body weight of 0.92 ± 0.01 g, were selected for this study. The juvenile fish were randomly distributed across 15 aquariums, each with a capacity of 325 L. Each aquarium measured 0.72 m in diameter and had a water depth of 0.8 m, housing 25 larvae per tank.

The experimental treatment was conducted over a duration of 60 days, utilizing five distinct alkalinity gradients: 0, 10, 20, 30, and 40 mmol/L. The concentration of carbonate alkalinity was determined based on the median lethal concentration (LC50) of red tilapia from our previous preliminary experiments, which was 59.49 mmol/L. The carbonate alkalinity of the water body was monitored using acid-base titration [29], and an appropriate amount of NaHCO_3_ was added to maintain the alkalinity at the target value. To regulate the alkalinity, 5 mmol/L of sodium bicarbonate (NaHCO_3_) was added bi-daily, and 50% of the water was replaced every two days to ensure optimal water quality. The experimental design was completely randomized, with each treatment group replicated three times. The alkalinity measurement was standardized through acid titration, expressed in millimoles per liter (mmol/L). During the course of the experiment, an air pump was utilized to ensure continuous aeration, thereby maintaining the water temperature at 28 ± 1 °C. The salinity and pH values of the five experimental groups ranged from 0 to 0.18‰ (PAL-SALT salinity meter, ATAGO, Fukaya, Japan) and from 7.24 to 8.10 (ST20 portable pH meter, OHAUS, Parsippany, NJ, USA), respectively. The water quality parameters were assessed on a weekly basis employing a pH meter, a dissolved oxygen meter, and a thermometer.

### 2.2. Collection of Experimental Samples

At the end of the experiment, three fish were randomly chosen from each treatment group. The selected samples were anesthetized using a concentration of 100 mg/L of MS-222 prior to dissection. During this procedure, the second gill arch was removed from the gill tissue and subsequently fixed in a 4% (*v*/*v*) paraformaldehyde solution. The fixed samples were then stored at 4 °C for histological analysis. Furthermore, additional gill tissues were harvested, rapidly frozen in liquid nitrogen, and preserved at −80 °C for biochemical and transcriptomic analyses.

### 2.3. Morphological Analysis of Gills

Paraffin-embedded sections of gill tissue were prepared using the hematoxylin–eosin staining method described by Hua et al. [2] for histological examination. The morphological characteristics of the gill tissues were quantified with ImageJ software (version 1.54d). The rate of deformation for the gill lamellae was determined by calculating the proportion of deformed lamellae in relation to the overall total lamellae.

Gill sections were subjected to staining by utilizing the enhanced TUNEL detection method, as established by Qiang et al. [30]. Apoptotic cells within each group were quantified with Image-Pro Plus 6.0 software.

### 2.4. Enzyme Activity Assays of Gills

After thawing the gill tissue samples on ice, approximately 0.1 g of each tissue was combined with 9 volumes of pre-cooled phosphate-buffered saline (PBS) and homogenized to create a 10% homogenate. The mixture was then centrifuged at 3000× *g* for 15 min, and the supernatant was collected. The supernatant was used to measure the activities of Na^+^-K^+^-2Cl^−^ Cotransporter 1 (NKCC1), Calcium/Magnesium-ATPase (Ca^2+^/Mg^2+^-ATP), Sodium/Potassium-ATPase (Na^+^/K^+^-ATP), superoxide dismutase (SOD), catalase (CAT), malondialdehyde (MDA), and glutathione peroxidase (GSH-PX). The methods of NKCC1, Ca^2+^/Mg^2+^-ATP, and Na^+^/K^+^-ATP were determined using an enzyme-linked immunosorbent assay (ELISA) in accordance with the method provided by the manufacturer. The activities of SOD, CAT, and MDA were conducted following the manufacturer’s instructions provided by the commercial kits (Jiancheng Biotech Co., Nanjing, China).

### 2.5. Transcriptome Sequencing of Gills

#### 2.5.1. RNA Extraction and Transcriptome Sequencing

Total RNA was isolated following the protocol provided with the RNA Purification Reagent (Invitrogen, Waltham, MA, USA). The RNA concentration and purity were assessed using a Nanodrop 2000 spectrophotometer (Thermo Fisher Scientific, Waltham, MA, USA). RNA integrity was evaluated via agarose gel electrophoresis, and the RNA Integrity Number (RIN) was quantified using an Agilent 2100 bioanalyzer (Agilent Technologies, Inc., Santa Clara, CA, USA).

Subsequent steps, including RNA purification, cDNA synthesis, library preparation, and sequencing, were conducted by Shanghai Personal Biotechnology Co., Ltd. (Shanghai, China) in according with the manufacturer’s instructions (Illumina, San Diego, CA, USA). The multiplexed libraries were generated with the Illumina TruSeqTM RNA sample preparation Kit (San Diego, CA, USA) and sequenced on the Illumina NovaSeq 6000 platform.

#### 2.5.2. Data Assembly and Identification of Differentially Expressed Genes

Reads of low quality were eliminated, which included those exhibiting adaptor contamination, empty reads, and low-quality sequences (reads containing unknown ‘N’ or shorter than 20 bp). Additionally, sequences shorter than 30 bp and those that were partially overlapping were also excluded. Then, the clean data were compared with the reference genome of *O.* spp. using Hisat2 (v2.0.1) [31].

To identify DEGs (differential expression genes), the expression level of each gene was calculated according to the expected number of fragments per kilobase of transcript sequence per million base pairs sequenced (FPKM) method. The expression levels of genes and transcripts were quantitatively analyzed by the software HTSeq (v2.0.1) [32]. Essentially, differential expression analysis was performed using the DESeq2 [33], and an absolute log2 (fold change) ≥ 1 and |*p*-adjust| ≤ 0.05 (DEGseq) were considered to be significantly DEGs. The DEGs were annotated with the methods that were similar to those mentioned above in terms of unigene annotation. Biological repetitive analysis was carried out to evaluate the quality of sequence data.

#### 2.5.3. Weighted Gene Co-Expression Network Analysis (WGCNA)

To investigate the relationships between genes, network analysis was conducted using WGCNA within the R package (http://www.genetics.ucla.edu/labs/horvath/CoexpressionNetwork/Rpackages/WGCNA, accessed on 7 September 2025) [34]. Then, we selected the appropriate soft threshold to construct the gene co-expression network. Gene modules were identified through hierarchical clustering analysis, and the characteristic genes of each module were determined. Additonally, we analyzed the correlation between the characteristic gene modules and “genotypes” across 15 samples. The correlations between groups and modules were represented by correlation coefficients that range from −1 to 1. Functional enrichment assessments of the module were conducted by GO and KEGG pathway analyses, using GOseq and KOBAS V2.0, respectively. Additionally, the interaction network of hub genes within the largest module was visualized using Cytoscape (V3.7.1) [35].

#### 2.5.4. Fuzzy C-Means Cluster Analysis

To categorize the differentially expressed genes according to their expression dynamics, fuzzy c-means clustering was applied. The analysis was carried out with the Mfuzz v3.9 package in R, utilizing the mean FPKM values [36]. The optimal configuration was identified as nine clusters, employing a fuzzification coefficient of 2.01.

#### 2.5.5. Quantitative Real-Time PCR (qPCR) Verification

To validate the Illumina sequencing data, 6 DEGs were selected for qPCR analysis using the same RNA samples for Illumina transcriptome profiling. The primers were designed with Prime Premier 5 software (Table S1), with the *β-actin* gene used as the internal reference for the qPCR analysis. The experimental protocols and analysis methods were outlined in our previously published study [37].

### 2.6. Statistical Analysis

All data were expressed as the mean and standard deviation (Mean ± SD). The statistical significance of the data was analyzed using Origin 2024b and SPSS 27.0. One-way ANOVA was conducted to assess the apoptosis rate of cells, antioxidant enzyme activities (SOD, CAT, MDA, GSH-Px), and the activities of NKCC1, Ca^2+^/Mg^2+^-ATPase, and Na^+^/K^+^-ATPase under different CA concentrations. Post hoc comparisons were performed using Duncan’s multiple range test with a significance level of *p* < 0.05. Additionally, repeated measures ANOVA (RM-ANOVA) was used to analyze the expression trends of qPCR genes across varying CA concentrations for statistical significance.

## 3. Results

### 3.1. Histopathological Observations

**Figure 1:**
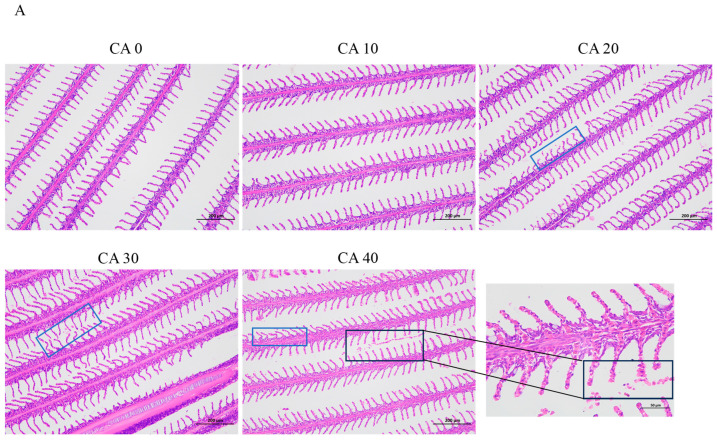

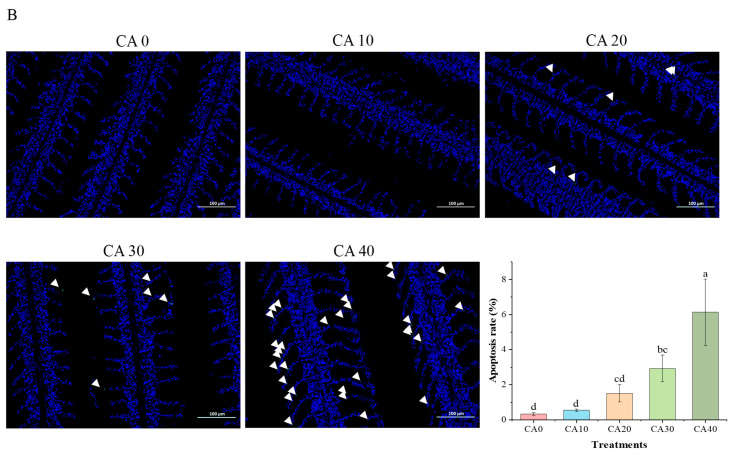
(**A**) Histological observations and measured parameters of gills of red tilapia under alkalinity stress (×100 and ×400 magnification). (**B**) TUNEL staining results of gill tissues (×200 magnification) and the apoptosis rate of gill cells. Apoptotic cells were denoted by white arrows. Different letters represent significant differences (*p* < 0.05) between different CA concentrations. The same letters indicate no significant differences among the CA concentration groups (*p* > 0.05).

As shown in Figure 1A, exposure to alkaline stress induced significant histopathological changes in the gill lamellae across the treatment groups. In the CA20 group, the gill lamellae exhibited marked deformation and curling (indicated by the blue square frame), which is indicative of early-stage tissue damage. Progressive deterioration was observed at higher concentrations, with the CA30 and CA40 groups displaying pronounced pathological features. Specifically, these groups showed marked gill tissue hyperplasia that was accompanied by numerous detached erythrocytes. Additionally, the gill lamellae appeared shortened and deformed, with enlarged tips (black square frame). Further morphological alterations included a thickening of the gill filaments, disorganized pillar cells, and enlarged, vacuolated chloride cells (black square frame), suggesting severe dysfunction in osmoregulatory and respiratory functions.

The one-way ANOVA showed that the concentration of CA had a significant effect on the apoptosis rate (Supplementary Table S4). An increase in apoptotic cell death and structural damage in the gill tissues was observed under CA-induced stress. As shown in Figure 1B (white arrows), the severity of these effects was positively correlated with increasing CA concentrations, a relationship confirmed by TUNEL staining. During the concentration range of CA10 to CA20, apoptotic cells (indicated by white arrows) were scattered throughout the entire filamentous epithelium. In the CA30 to CA40 group, continuous apoptotic regions were observed. The histomorphometric analysis revealed that the apoptotic index rose from 0.33 ± 0.08 in the controls to 6.14 ± 1.89 in the CA40 group (*p* < 0.05, one-way ANOVA). This apoptotic surge paralleled the observed histopathological changes, suggesting a mechanistic link between programmed cell death and tissue remodeling under chronic alkalinity stress.

### 3.2. Enzymatic Activities Assays

**Figure 2:**
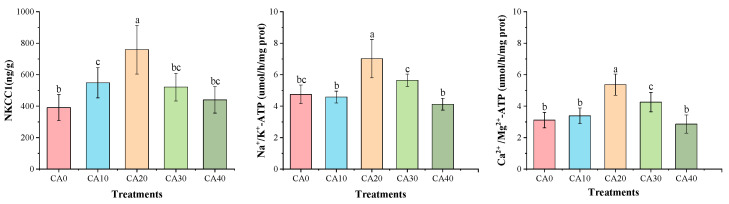
Gill activities of energy metabolism in red tilapia among different alkalinity concentration groups (mean ± SD, n = 9). Different letters represent significant differences (*p* < 0.05) between different CA concentrations. The same letters indicate no significant differences among the CA concentration groups (*p* > 0.05).

**Figure 3:**
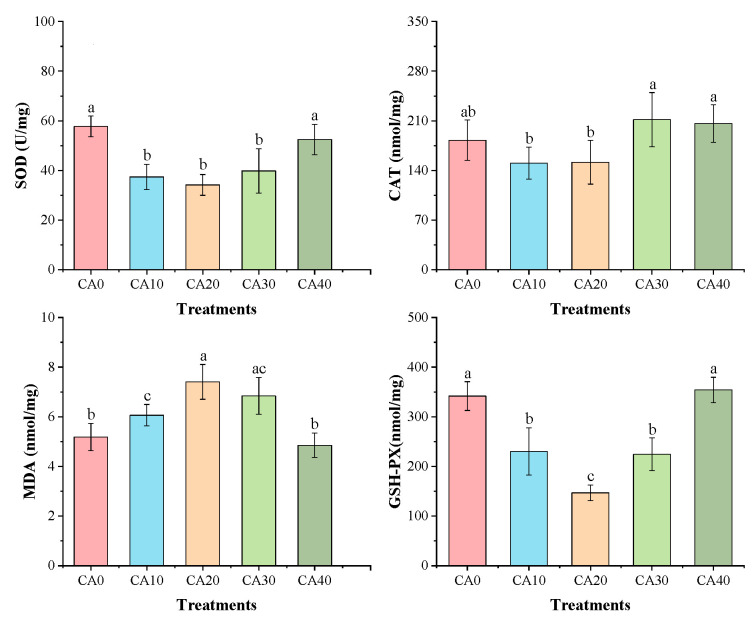
Gill activities of antioxidant enzymes in red tilapia among different alkalinity concentration groups (mean ± SD, n = 9). Different letters represent significant differences (*p* < 0.05) between different CA concentrations. The same letters indicate no significant differences among the CA concentration groups (*p* > 0.05).

The one-way ANOVA showed that the concentration of CA had a significant effect on the enzyme activities related to energy metabolism and antioxidant capacity in red tilapia (Supplementary Table S4). Alkalinity stress significantly influenced the energy metabolism and antioxidant capacity of red tilapia (Figure 2 and Figure 3). The activities of NKCC1, Na^+^/K^+^-ATPase, and Ca^2+^/Mg^2+^-ATPase followed similar trends, initially increasing and then decreasing as the alkalinity concentrations rose. The highest values for all three enzymes were observed in the CA20 treatment, showing significant differences compared to other treatments (*p* < 0.05).

In contrast, the enzymatic activities of SOD, CAT, and GSH-Px were significantly lower in the CA20 treatment under alkalinity stress than in the control group (*p* < 0.05). Conversely, MDA activity exhibited an inverse trend, peaking in the CA20 group and differing significantly from treatments CA0, CA10, and CA40 (*p* < 0.05).

### 3.3. Overview of Sequencing Data

The Illumina RNA-seq generated 678,058,696 raw reads from the 12 tested gill tissue samples. After removing the low-quality reads, 33,894 unigenes were obtained. The quality evaluation analysis showed that the Q20 and Q30 values of each sample exceeded 98.50% and 95.73%, respectively, and the GC contents were both above 43.68% (Table S2). The results indicate that all tested samples met the established high-quality standards. To identify putative functions, all the unigenes were blasted against five protein databases for annotation analysis, including NR, SwissProt, KEGG, GO, and NOG (Table S3). The NR database provided the highest number of gene annotations (30,732, 90.67%), followed by the NOG (27,846, 82.15%) and the SwissProt databases (25,274, 74.56%). The KEGG databases yielded the lowest unigene annotations (16,225, 47.86%) compared to the other four databases.

**Figure 4:**
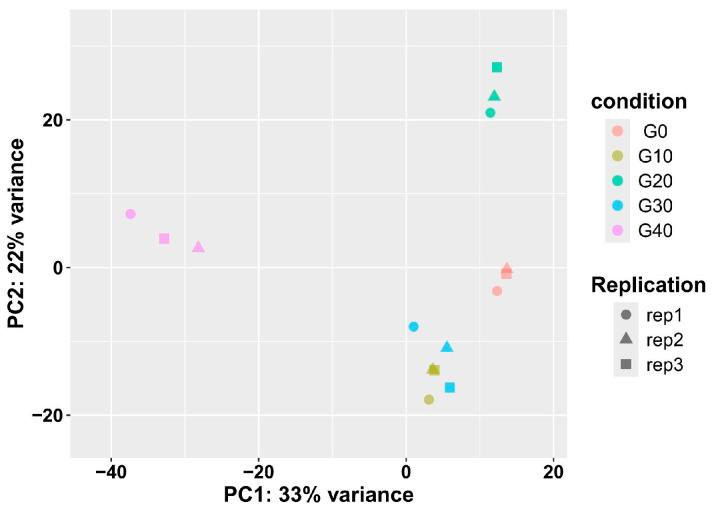
Principal component analysis (PCA) plots showing distribution of samples from five groups.

To evaluate the biological variability, the Pearson correlation coefficient was calculated for the biological replicates, revealing highly consistent results for CA exposure in this study (Table S7). Principal component analysis (PCA) further indicated that the replicates of each group had highly repeatable expression (Figure 4). For instance, Figure 4 demonstrated a significant separation between the CA40 and CA20 groups, while Table S7 showed that the correlation values between the CA40 and CA20 groups are consistently lower than those observed within other groups.

### 3.4. Analysis of the Differentially Expressed Genes

**Figure 5:**
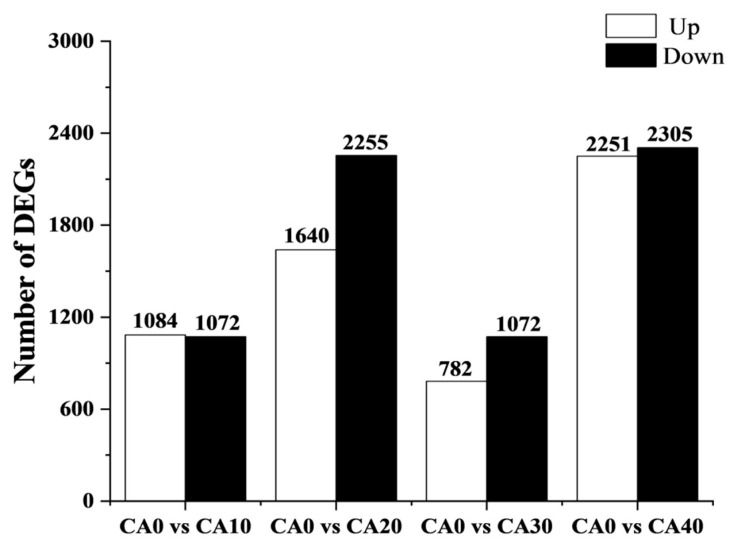
The number of differentially expressed genes identified in each treatment group compared to the control group. The white bars represent the number of significantly upregulated DEGs, while the black bars represent the number of significantly downregulated DEGs.

Differential gene expression analysis across the five treatment groups was conducted using pairwise comparisons (Figure 5). Compared to the control, 2156 differentially expressed genes (DEGs) (1084 upregulated and 1072 downregulated genes) were found in the CA10 group, 3895 DEGs (1640 upregulated and 2255 downregulated genes) in the CA20 group, 1854 DEGs (782 upregulated and 1072 downregulated genes) in the CA30 group, and 4556 DEGs (2251 upregulated and 2305 downregulated genes) in the CA20 group were identified.

### 3.5. Gene Co-Expression Network Analysis

**Figure 6:**
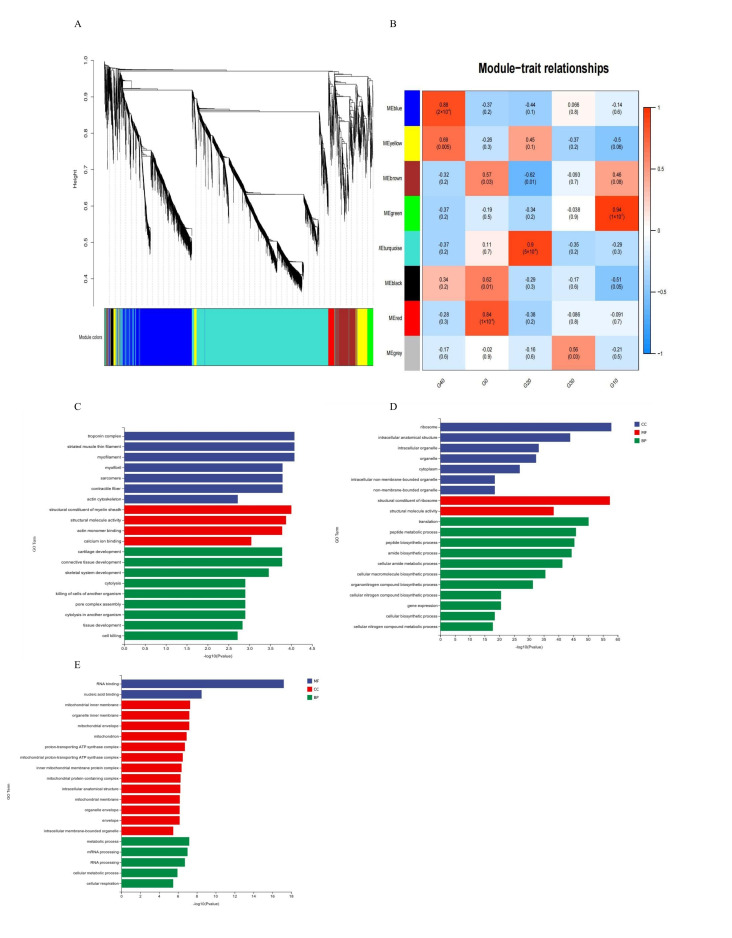
A functional module within the gill transcriptome was identified through Weighted Gene Correlation Network Analysis (WGCNA). (**A**) resents the gene dendrogram, which was generated by clustering genes based on a consensus topological overlap measure of dissimilarity. In this clustering, 40 distinct modules were detected, with each color representing a unique module. The associations between these modules and experimental groups are illustrated in (**B**). Furthermore, Gene Ontology (GO) enrichment analyses were separately conducted for the CA10, CA20, and CA40 groups, with the corresponding results displayed in panels (**C**), (**D**), and (**E**), respectively.

WGCNA systematically uncovers correlation patterns in gene expression data and identifies the key gene modules and regulatory hubs that are associated with specific phenotypes. Through the establishment of a gene co-expression network, genes demonstrating comparable expression behaviors were clustered into distinct modules, uncovering collaborative regulatory interactions. A total of 40 modules were identified via WGCNA (Figure 6A). The correlation analysis showed that module blue-G40, module green-G10, and module turquoise-G20 were significantly correlated with phenotypes (exposure to different CA concentrations in the present study) (Figure 6B). According to GO enrichment analysis, the CA10 group showed significant enrichment in biological functions such as the troponin complex, structural composition of the myelin sheath, and cartilage development (Figure 6C). CA20 was enriched in ribosomes and structural constituents of the ribosome (Figure 6D), while CA40 was enriched in the RNA binding (Figure 6E). In the KEGG pathway enrichment analysis, CA10 was enriched in the focal adhesion (Figure S1A), while CA20 was enriched in the ribosome (Figure S1B). Spliceosome, Oxidative phosphorylation, and the Citrate cycle (TCA cycle) were the main enriched signaling pathways in the CA40 group (Figure S1C).

### 3.6. Correlation of Gene Expression Patterns with the CA Concentration

**Figure 7:**
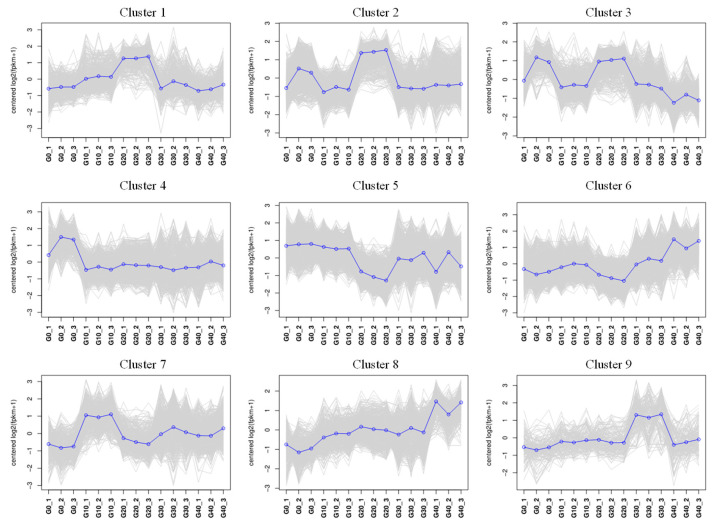
The fuzzy analysis revealed twelve distinct patterns of gene expression. The concentration of CA is shown on the x-axis, while the y-axis displays the log 2-transformed FPKM values.

Gene expression patterns arising from exposure to various concentrations of CA in water were depicted through fuzzy c-means cluster analysis. A total of nine clusters were identified according to the FPKM values. Of these clusters, clusters 3 and 4 indicated a reduction in gene expression with rising CA concentration, whereas clusters 6 and 8 demonstrated a positive correlation with the CA concentration in gene expression (Figure 7).

**Figure 8:**
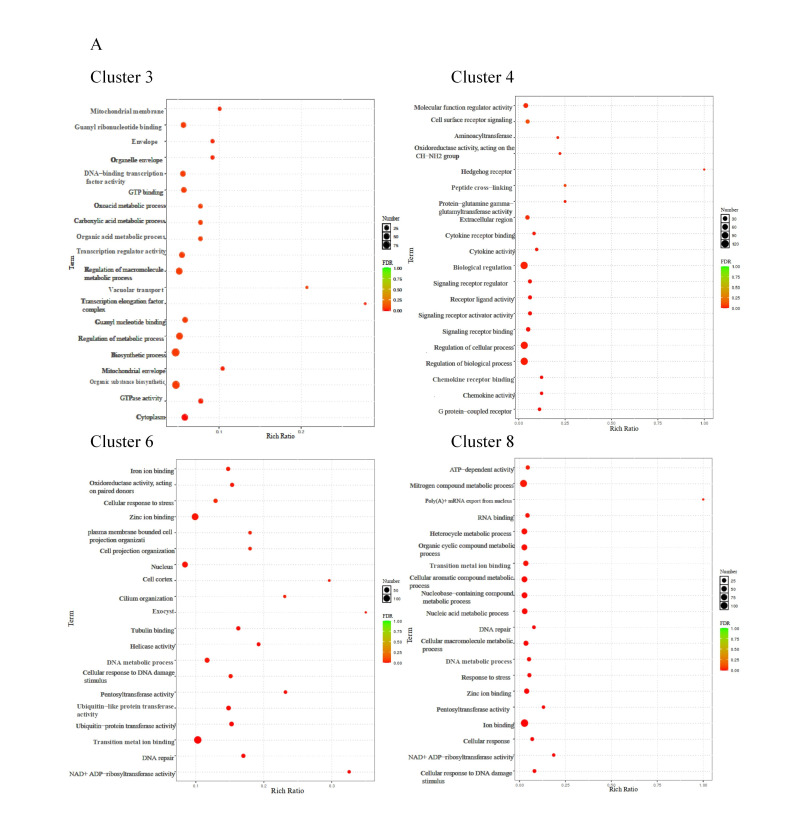

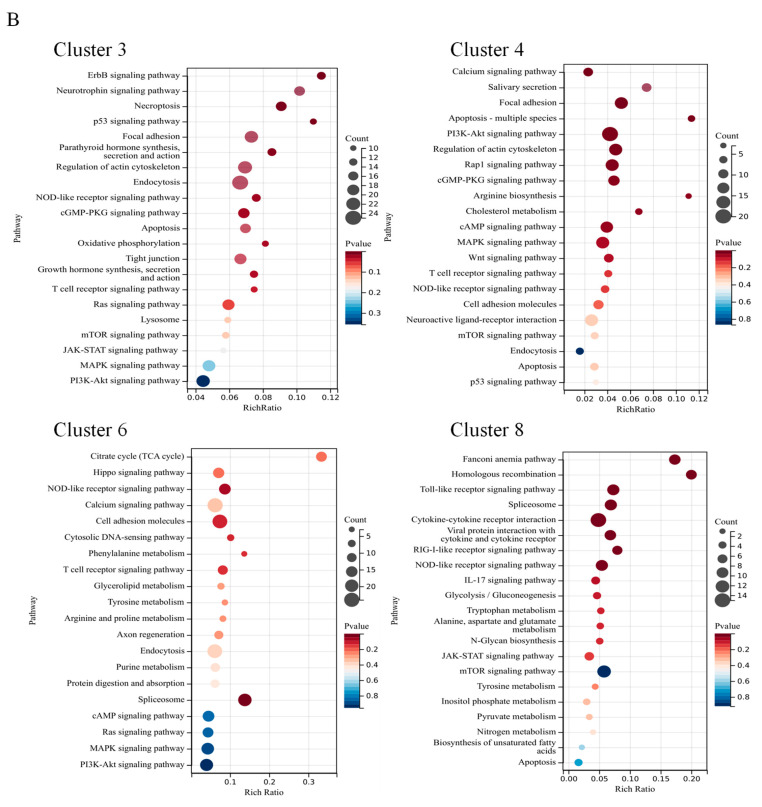

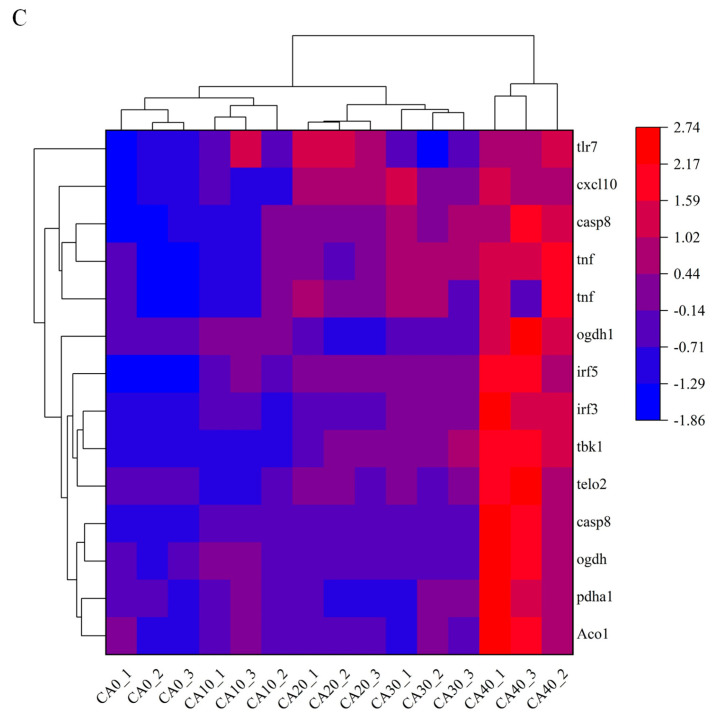
The functional examination of clusters 3, 4, 6, and 8 identified through fuzzy analysis. (**A**) The Gene Ontology (GO) enrichment assessment of genes within the affected clusters by CA. (**B**) The Kyoto Encyclopedia of Genes and Genomes (KEGG) pathway enrichment assessment of genes influenced by CA. (**C**) A heatmap depicting the transcriptional expression of genes associated with the KEGG category related to metabolism and the immune system.

The GO enrichment results indicated that clusters 3 and 4 were associated with biological functions, such as the regulation of macromolecule metabolic process, organic substance biosynthetic process, and other cellular activities. In cluster 6 and cluster 8, significant enrichments were observed for terms, including a cellular response to stress, ion binding, nitrogen compound metabolic process, ATP-dependent activity, and various metabolic functions (Figure 8A). Meanwhile, the KEGG pathways analysis of exhibiting a negative correlation with CA concentration (cluster 3 and cluster 4) revealed enrichments in endocytosis, focal adhesion, PI3K−Akt signaling pathway, MAPK signaling pathway, and other related pathways. Cluster 6 was enriched in cell adhesion molecules, the Citrate cycle (TCA cycle), NOD−like receptor signaling pathway, and other pathways. While cluster 8 was mainly enriched in a cytokine–cytokine receptor interaction, toll-like receptor signaling pathway, and mTOR signaling pathway (Figure 8B). Gene expression linked to the metabolic and immune processes, which were enriched in clusters 6 and 8, is shown in a heatmap (Figure 8C). These genes showed an increasing trend with increasing CA concentrations.

### 3.7. qPCR Verification

**Figure 9:**
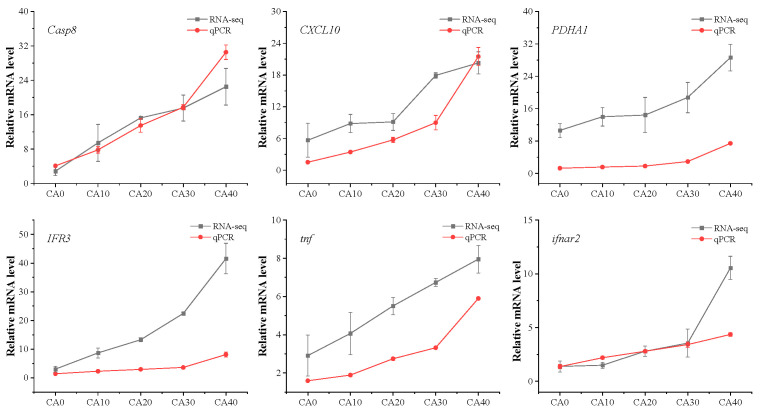
qPCR and RNA-seq results of *Casp8*, *CXCL10*, *PDHA1*, *IFR3*, *tnf,* and *ifnar2* in the gill of tilapia from different CA concentrations.

In this investigation, our attention was directed towards the molecular toxicological mechanisms through which CA impacts the metabolic and immune systems of red tilapia. Accordingly, we conducted an analysis of six randomly chosen genes associated with the KEGG pathway for metabolism and the immune system, which was confirmed through qPCR. Repeated measures ANOVA showed that CA concentration had a significant effect on *Casp8*, *CXCL10*, *PDHA1*, *IFR3*, *tnf*, and *ifnar2* (Supplementary Table S5). Consistent with the RNA-seq findings (Figure 9), the qPCR results also revealed elevated expression levels of these genes as the CA concentrations increased.

## 4. Discussion

The utilization of saline–alkali water resources can not only alleviate the pressure caused by excessive aquaculture but also enhance the economic benefits of local communities. Therefore, in recent years, an increasing number of researchers have focused on the attention to the adaptation and tolerance of fish to alkaline water. Up to now, various types of fish have been reported to exhibit tolerance to alkaline conditions, such as *Oreochromis niloticus* [17], *Cyprinus carpio* *Songpu,* and the spotted sea bass, which have a certain tolerance to alkaline waters [38,39]. Hua et al. showed that *Micropterus salmoides* die in water with an alkalinity of 32.5 mmol/L, with a 96-h LC50 of 41.41 mmol/L. These findings indicated that *M. salmoides* has a strong tolerance to alkali conditions [2]. A previous study revealed that the 96 h LC50 at 96 h for tilapia is 4.639 g/L, suggesting that this species could be valuable for aquaculture with alkaline water if it can adapt successfully to such conditions. To investigate the effects of saline water on the physiological conditions and tissue structure of red tilapia, a long-term chronic salinization stress experiment lasting 60 days was carried out.

Gills function as respiratory organs for fish and various aquatic species, facilitating gas exchange, osmoregulation, and excretion. In their research, Shan et al. discovered that in Amur ide, the gills adapted to alkaline environments, and exhibited broad gill lamellae, elongated secondary gill lamellae, significant spacing between secondary gill lamellae, an abundance of mitochondria-rich cells, as well as mucous cells enveloping the gill filament epithelium, and have robust pavement cells and a canal of which effectively hinder toxic substances from penetrating the bloodstream [40]. These findings are consistent with our study on the histopathology of gills in red tilapia. Our findings reveal that gill deformation rates correlated with the length of exposure to alkali stress. The most pronounced gill deformation occurred at a treatment level of 40 mmol/L alkalinity, indicating that exposure to high alkalinity resulted in gill remodeling. In comparison to the control group, the gill lamellae in the CA40 group were found to be thicker and shorter, with the gill filaments also exhibiting increased thickness. Such alterations may signify gill remodeling as a response to the alkalinity stress observed in red tilapia. A similar result was also reported in *M. salmoides* by Hua et al. [2]. In addition, the gills of the CA40 group exhibited a reduced number of interlamellar cell masses. This morphological change likely impedes the penetration of hypertonic ions.

Our results indicate that moderate alkalinity (CA20) induces a compensatory upregulation of antioxidant enzymes to counteract rising ROS levels, as evidenced by the peak in enzyme activity. However, excessive alkalinity (CA40) likely exceeds the detoxification capacity, leading to structural damage in gill tissues (as shown by the histopathological analysis), which impairs enzyme synthesis and secretion. The current results demonstrate that antioxidant activities were elevated under high alkali stress, while the activities of ion transport-related enzymes were inhibited. When aquatic animals experience environment stress, such as high alkali stress, respiratory depression can lead to the production of numerous reactive oxygen species (ROS) [41,42]. Excess or residual ROS may trigger oxidative stress defense mechanisms, resulting in increased levels of antioxidant enzymes, including superoxide dismutase (SOD), catalase (CAT), and glutathione peroxidase (GPX) [43]. However, this rapid response mechanism may become imbalanced over time; prolonged exposure to alkaline stress can lead to the continuous accumulation of ROS, causing oxidative damage [44]. SOD catalyzes the disproportionation of superoxide anion radicals (O_2_^•−^) into hydrogen peroxide (H_2_O_2_) and molecular oxygen (O_2_) [45]. Conversely, GPX facilitates the reaction between GSH and H_2_O_2_, converting them into water and glutathione disulfide (GSSG), thereby protecting cells from oxidative damage [46]. As a crucial reducing agent, GSH compensates for the limitations of SOD and GPX in scavenging hydroxyl radicals [47]. Under high alkali stress, elevated ROS levels also result in mitochondrial oxidative damage and impaired aerobic respiration [48]. The findings of this study align with these observations. With increasing CA stress concentration, the metabolic capacity of red tilapia diminishes, the ion exchange capacity of gill tissues decreases, and oxidative activity significantly rises to adapt to external environmental stresses.

In this study, transcriptome analyses were conducted for the gill tissues of red tilapia under different concentrations of alkalinity to explore the molecular mechanism of its response. The differential gene expression analysis revealed that the CA20 and CA40 treatments induced the most substantial transcriptional changes compared to the control. Specifically, 3895 DEGs were identified under the CA20 conditions (1640 upregulated and 2255 downregulated), while the CA40 conditions resulted in 4556 DEGs (2251 upregulated and 2305 downregulated). We propose that the heightened carbonate alkalinity in these groups activated distinct stress-response pathways, triggering a more extensive transcriptomic reprogramming. The results of the GO and KEGG analyses revealed that the DEGs were mainly enriched in signaling pathways that are related to metabolism and immunity, including the Citrate cycle (TCA cycle), NOD−like receptor signaling pathway, PI3K−Akt signaling pathway, MAPK signaling pathway, and mTOR signaling pathway. Previous studies showed that disease resistance would be attenuated [49,50] and the inhibitory effect on the immune system of fish due to the stress of the external environment [51,52]. In addition, the biological processes such as phagocytosis and natural killer cell activity were also impaired during these stress conditions [53]. Therefore, the significant enrichment of immunity-related pathways observed in this study is consistent with these findings.

The tricarboxylic acid (TCA) cycle is a fundamental metabolic pathway that integrates carbon metabolism, electron transport, and oxidative phosphorylation, playing a crucial role in redox reactions [54]. Upon exposure to CA, the activities of four key TCA cycle enzymes—isocitrate dehydrogenase (IDH), α-ketoglutarate dehydrogenase (KDH), succinyl-CoA ligase, and malate dehydrogenase (MDH)—were downregulated, along with the expression levels of six other enzymes involved in the cycle. Consequently, this downregulation of these enzymes led to the accumulation of four differentially expressed metabolites (DMs): α-ketoglutarate, citrate, cis-aconitate, and phosphoenolpyruvate. These findings align with a recent study reporting elevated malate levels under CA stress [55].

Simultaneously, the MAP-activated protein kinase (MAPK) signaling pathway acts as a central metabolic regulator, coordinating cellular physiology by modulating energy homeostasis, glucose protein metabolism, and cell growth. Functioning as an energy rheostat, MAPK maintains metabolic balance by suppressing anabolic suppression (reducing ATP consumption) while activating catabolic pathways (enhancing ATP production) [56,57]. Beyond its role in energy sensing, MAPK regulates diverse physiological processes, including hepatic lipid homeostasis [58,59], metabolic regulation [60], anti-inflammatory actions [32], and longevity modulation [61].

Under alkaline stress, disruptions in ion homeostasis and metabolic equilibrium increase ATP demand [62]. As a critical energy sensor, MAPK helps maintain redox homeostasis by fine-tuning ATP production and directly regulating oxidative stress defense mechanisms through key signaling pathways and transcription factors. This mechanistic insight explains why the 14 DEGs expression levels of those identified in this study exhibited upregulated expression in response to increasing CA concentrations.

## 5. Conclusions

In this study, red tilapia (*O.* spp.) exhibited high tolerance to alkaline conditions. However, severe alkalinity (40 mmol/L, CA40 group) caused significant histopathological damage to gill tissue, as confirmed by the physiological and histological analyses. Alkaline stress also suppressed antioxidant activity while enhancing energy metabolism, indicating an elevated metabolic demand for stress adaptation. The RNA-seq analysis revealed the upregulation of differentially expressed genes (DEGs) associated with key metabolic and immune pathways, including the Citrate cycle (TCA cycle), NOD−like receptor signaling pathway, PI3K−Akt signaling pathway, MAPK signaling pathway, and mTOR signaling pathway. These findings provide mechanistic insights into the regulatory response of red tilapia to alkalinity stress, as well as theoretical recommendations for the selection and breeding of new alkalinity-tolerant strains.

## Data Availability

All of the data are included in the article.

## Supplementary Materials

The following supporting information can be downloaded at: https://www.mdpi.com/article/10.3390/antiox14091112/s1, Figure S1: KEGG pathway enrichment analysis of functional modules in the gill transcriptome; Table S1: Primers used for qPCR verification; Table S2: Summary of the transcriptome sequencing data; Table S3: Summary of the annotation statistics of the unigenes of the transcriptome; Table S4: One-way ANOVA analysis of variance for the CA concentrations (0, 10, 20, 30 and 40 mmol/L) on enzyme activity assays of gill; Table S5: Analysis of significant trends in RM-ANOVA for Genes in qPCR under different CA concentrations; Table S6: The signaling pathways enriched by differentially expressed genes (DEGs) in the 4 Clusters, along with their *p*-values; Table S7: Correlation coefficient table for each samples.
