# Supplementary material for: Physiological and Transcriptome Analyses Offer Insights into Revealing the Mechanisms of Red Tilapia (Oreochromis spp.) in Response to Carbonate Alkalinity Stress
Source: Antioxidants (Basel). 2025 Sep 13;14(9):1112. doi: 10.3390/antiox14091112 (PMC12466869; doi:10.3390/antiox14091112)

Supplementary Table S1. Primers used for qPCR verification

Target gene	Sequence (F:5'-3')	Sequence (R:5'-3')
PDHA1	GTCCTATGCGTCAGAGAGGC	CTGAATCTCCTCGCGGGTAC
IRF3	GCAGACAGCCCCAAGAGATT	TTGTGGGTCAGGCCACTTTT
Casp8	GCAAGCCCAAAGTGTTCCTG	CATCAGCTTCCTCGGGGATC
tnf	CCAACGCTGGTCTCACTCAT	GTTCTCAGTCTGTCCCCAGC
CXCL10	CGTTGCCTGCTTGCTTATCC	GGCTCAGCCTTGATGACCTT
Ifnar2	GGACAGCCACACGGACTTAA	CGCAGAGTAGGGCATATGCA
β-actin	GTACCACCATGTACCCTGGC	TGAAGTTGTTGGGCGTTTGG

Supplementary Table S2. Summary of the transcriptome sequencing data.

Sample	Raw Data	Clean Data	N (%)	GC (%)	Q20 (%)	Q30 (%)	Total_Mapped (%)
CA0_1	40340844	39859470	0.002379	44.79	98.51	95.73	84.84
CA0_2	38943354	38552700	0.002367	45.72	98.60	95.89	87.18
CA0_3	35940162	35557052	0.002395	45.43	98.57	95.86	86.74
CA10_1	46440754	45942784	0.002404	45.11	98.60	95.96	84.94
CA10_2	45269934	44793056	0.002416	44.83	98.67	96.16	84.91
CA10_3	46092538	45497282	0.000816	45.12	99.12	96.74	83.78
CA20_1	48212624	47647766	0.000821	45.22	99.19	96.93	86.94
CA20_2	40252402	39828988	0.002392	45.57	98.60	95.92	87.17
CA20_3	54502448	53723166	0.001545	45.97	98.88	96.93	88.62
CA30_1	50572196	49874112	0.001525	45.20	98.90	97.05	85.62
CA30_2	46431376	45628020	0.006654	45.07	98.68	96.51	83.96
CA30_3	52371328	51533802	0.006689	44.64	98.72	96.61	85.22
CA40_1	50717232	49793804	0.005263	43.68	98.60	96.37	80.60
CA40_2	40479188	39651534	0.001505	44.40	98.54	96.26	82.58
CA40_3	41492352	40676116	0.001619	43.85	98.50	96.14	81.25

Supplementary Table S3. Summary of the annotation statistics of the unigenes of the transcriptome.

**Database**	**Number of Annotated Unigenes**	**Percentage (%)**
Annotated in NR	30732	90.67
Annotated in SwissProt	25274	74.56
Annotated in KEGG	16225	47.86
Annotated in NOG	27846	82.15
Annotated in GO	19259	56.82

Supplementary Table S4. One-way ANOVA analysis of variance for the CA concentrations (0, 10, 20, 30 and 40 mmol/L) on enzyme activity assays of gill. df, degrees of freedom; *F*, mean square; Sig. p-value.

	Source	df	*F*	Sig.
NKCC1	CA concentration	4	174552.37421	<0.0001
	Errow	40	10909.19921	
Ca^{2+}/Mg^{2+}-ATP	CA concentration	4	9.45193	<0.0001
	Errow	40	0.33218	
Na^{+}/K^{+}-ATP	CA concentration	4	11.78116	<0.0001
	Errow	40	0.45493	
SOD	CA concentration	4	938.06477	<0.0001
	Errow	40	35.46172	
CAT	CA concentration	4	7579.88374	<0.0001
	Errow	40	882.65618	
MDA	CA concentration	4	10.45892	<0.0001
	Errow	40	0.35367	
GSH-PX	CA concentration	4	68445.03721	<0.0001
	Errow	40	1015.49984	
Apoptotic rate	CA concentration	4	17.00068	1.06574×10^{-4}
	Errow	10	0.87886	

Supplementary Table S5. Analysis of significant trends in RM-ANOVA for Genes in qPCR under different CA concentrations. df, degrees of freedom; *F*, mean square; Sig. p-value.

Sourse	CA concentration	Gene name	Type III Sum of Squares	df	*F*	Sig
CA concentration	Linear	*Casp8*	1821.937	1	318.417	<0.001
		CXCL10	1052.963	1	161.663	<0.001
		PDHA1	441.763	1	120.393	<0.001
		IRF3	1671.609	1	244.890	<0.001
		tnf	77.944	1	303.141	<0.001
		ifnar2	113.980	1	3138.505	<0.001

Supplementary Table S6. The signaling pathways enriched by differentially expressed genes (DEGs) in the 4 Clusters, along with their *P*-values.

	Pathway	*P*_value
Cluster 3	ErbB signaling pathway	0.000389
	Neurotrophin signaling pathway	0.000406
	Necroptosis	0.001462
	p53 signaling pathway	0.002269
	Focal adhesion	0.006122
	Parathyroid hormone synthesis, secretion and action	0.006528
	Regulation of actin cytoskeleton	0.009285
	Endocytosis	0.009697
	NOD-like receptor signaling pathway	0.016931
	cGMP-PKG signaling pathway	0.019405
	Apoptosis	0.020115
	Oxidative phosphorylation	0.020615
	Tight junction	0.022003
	Growth hormone synthesis, secretion and action	0.023917
	T cell receptor signaling pathway	0.035676
	Ras signaling pathway	0.0582
	Lysosome	0.13254
	mTOR signaling pathway	0.133119
	JAK-STAT signaling pathway	0.179384
	MAPK signaling pathway	0.244066
	PI3K-Akt signaling pathway	0.355593

Cluster 4	Calcium signaling pathway	0.001358
	Salivary secretion	0.001779
	Focal adhesion	0.002395
	Apoptosis - multiple species	0.003138
	PI3K-Akt signaling pathway	0.005611
	Regulation of actin cytoskeleton	0.006054
	Rap1 signaling pathway	0.010915
	cGMP-PKG signaling pathway	0.017082
	Arginine biosynthesis	0.023051
	Cholesterol metabolism	0.026916
	cAMP signaling pathway	0.032382
	MAPK signaling pathway	0.054967
	Wnt signaling pathway	0.062615
	T cell receptor signaling pathway	0.120783
	NOD-like receptor signaling pathway	0.129093
	Cell adhesion molecules	0.173103
	Neuroactive ligand-receptor interaction	0.329471
	mTOR signaling pathway	0.336796
	Endocytosis	0.866759
	Apoptosis	0.325321
	p53 signaling pathway	0.402153
Cluster 6	Citrate cycle (TCA cycle)	0.210969
	Hippo signaling pathway	0.212117
	NOD-like receptor signaling	0.055996

	pathway	
	Calcium signaling pathway	0.345141
	Cell adhesion molecules	0.107922
	Cytosolic DNA-sensing pathway	0.114123
	Phenylalanine metabolism	0.124444
	T cell receptor signaling pathway	0.126437
	Glycerolipid metabolism	0.277108
	T cell receptor signaling pathway	0.126437
	Tyrosine metabolism	0.260376
	Arginine and proline metabolism	0.260403
	Axon regeneration	0.266277
	Endocytosis	0.374804
	Purine metabolism	0.408472
	Protein digestion and absorption	0.432369
	Spliceosome	0.000175
	cAMP signaling pathway	0.855465
	Ras signaling pathway	0.869859
	MAPK signaling pathway	0.909803
	PI3K-Akt signaling pathway	0.958112
Cluster 8	Fanconi anemia pathway	6.24E-07
	Homologous recombination	8.41E-07
	Toll-like receptor signaling pathway	0.000349
	Spliceosome	0.000549
	Cytokine-cytokine receptor interaction	0.001044

Viral protein interaction with cytokine and cytokine receptor	0.001126
RIG-I-like receptor signaling pathway	0.001658
NOD-like receptor signaling pathway	0.003311
IL-17 signaling pathway	0.071251
Glycolysis / Gluconeogenesis	0.088514
Tryptophan metabolism	0.101015
Alanine, aspartate and glutamate metabolism	0.105066
N-Glycan biosynthesis	0.10918
JAK-STAT signaling pathway	0.134956
mTOR signaling pathway	0.917619
Tyrosine metabolism	0.228135
Inositol phosphate metabolism	0.318521
Pyruvate metabolism	0.322955
Nitrogen metabolism	0.391249
Biosynthesis of unsaturated fatty acids	0.599245
Apoptosis	0.710833

Supplementary Table S7. Correlation coefficient table for each samples.

**Sample**	**G0_1**	**G0_2**	**G0_3**	**G10_1**	**G10_2**	**G10_3**	**G20_1**	**G20_2**	**G20_3**	**G30_1**	**G30_2**	**G30_3**	**G40_1**	**G40_2**	**G40_3**
**G0_1**	1	0.96	0.98	0.99	0.99	0.97	0.97	0.94	0.94	0.97	0.97	0.98	0.93	0.99	0.95
**G0_2**	0.96	1	0.99	0.96	0.95	0.92	0.92	0.97	0.97	0.93	0.95	0.96	0.84	0.93	0.86
**G0_3**	0.98	0.99	1	0.98	0.98	0.96	0.94	0.97	0.97	0.96	0.96	0.98	0.89	0.97	0.91
**G10_1**	0.99	0.96	0.98	1	1.00	0.96	0.98	0.94	0.94	0.96	0.98	0.99	0.91	0.98	0.93
**G10_2**	0.99	0.95	0.98	1.00	1	0.97	0.97	0.94	0.94	0.97	0.98	0.98	0.92	0.99	0.94
**G10_3**	0.97	0.92	0.96	0.96	0.97	1	0.93	0.93	0.94	0.99	0.93	0.95	0.97	0.98	0.98
**G20_1**	0.97	0.92	0.94	0.98	0.97	0.93	1	0.89	0.88	0.93	0.98	0.97	0.88	0.96	0.91
**G20_2**	0.94	0.97	0.97	0.94	0.94	0.93	0.89	1	1.00	0.94	0.92	0.94	0.87	0.93	0.89
**G20_3**	0.94	0.97	0.97	0.94	0.94	0.94	0.88	1.00	1	0.94	0.92	0.94	0.88	0.93	0.90
**G30_1**	0.97	0.93	0.96	0.96	0.97	0.99	0.93	0.94	0.94	1	0.94	0.95	0.96	0.98	0.97
**G30_2**	0.97	0.95	0.96	0.98	0.98	0.93	0.98	0.92	0.92	0.94	1	0.98	0.87	0.96	0.89
**G30_3**	0.98	0.96	0.98	0.99	0.98	0.95	0.97	0.94	0.94	0.95	0.98	1	0.90	0.97	0.92
**G40_1**	0.93	0.84	0.89	0.91	0.92	0.97	0.88	0.87	0.88	0.96	0.87	0.90	1	0.97	1.00
**G40_2**	0.99	0.93	0.97	0.98	0.99	0.98	0.96	0.93	0.93	0.98	0.96	0.97	0.97	1	0.98
**G40_3**	0.95	0.86	0.91	0.93	0.94	0.98	0.91	0.89	0.90	0.97	0.89	0.92	1.00	0.98	1

Supplementary Figure S1. KEGG pathway enrichment analysis of functional modules in the gill transcriptome.

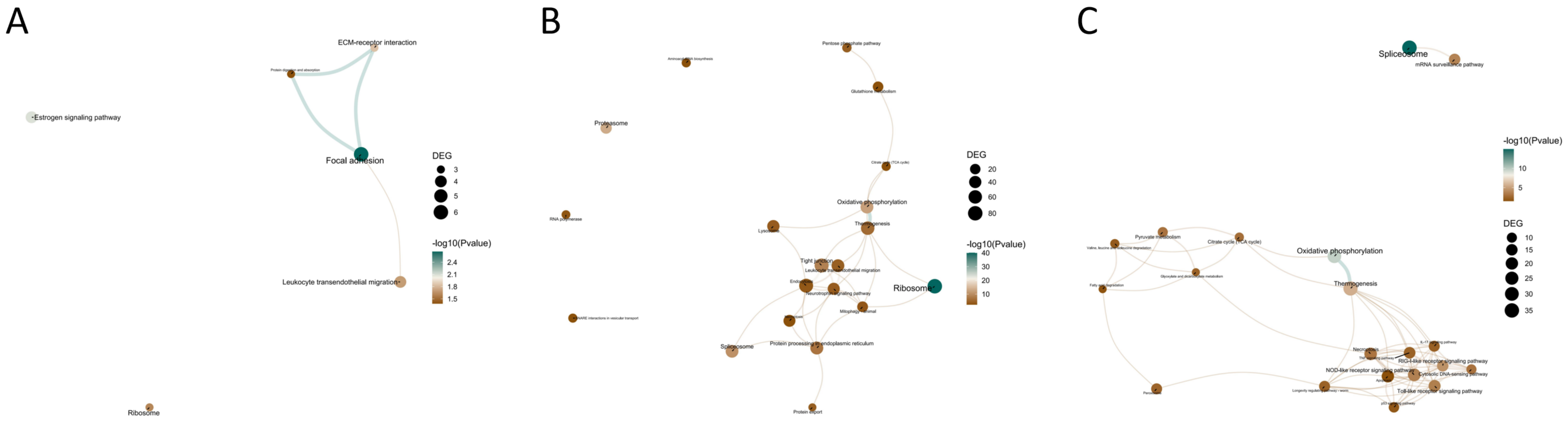

Supplement: Supplementary file 1 [file antioxidants-14-01112-s001.zip › antioxidants-3800437-supplementary.pdf]
